# Non-kin caregivers of terminally ill people: Contributions, experiences, and needs: A protocol for a mixed-methods study

**DOI:** 10.1371/journal.pone.0306282

**Published:** 2024-06-27

**Authors:** Maria Heckel, Franziska A. Herbst

**Affiliations:** 1 Department of Palliative Medicine, CCC Erlangen–EMN, Universitätsklinikum Erlangen, Friedrich-Alexander-Universität Erlangen-Nürnberg (FAU), Erlangen, Germany; 2 Institute for General Practice and Palliative Care, Hannover Medical School, Hannover, Germany; PLOS: Public Library of Science, UNITED KINGDOM

## Abstract

**Background:**

The role of non-kin caregivers, such as friends, neighbours, and acquaintances, in providing end-of-life care is significant but often overlooked in research and policy discussions. These caregivers provide extensive support for individuals in end-of-life care, in addition to or instead of family members. However, there is limited evidence in the literature regarding the experiences, burdens, and benefits of non-kin caregivers.

**Aims:**

The aim of this research is to examine the role and contributions of non-kin caregivers in end-of-life care. The study intends to uncover their experiences, associated challenges, benefits, and requirements for support.

**Methods:**

In order to achieve this objective, a mixed-methods approach will be employed, gathering data through structured questionnaires from approximately 150 non-kin caregivers and in-depth interviews with up to 25 participants. The questionnaires will measure the impact, burden, and benefits of caregiving. The Burden Scale for Family Caregivers, the Benefits of Being a Caregiver Scale, the Family Inventory of Needs, the Positive Mental Health Scale, a Graphic Closeness Scale, and selected items of the Eurofamcare Common Assessment Tool for socio-demographic and caregiving-related data will be used. Quantitative data will be analysed using IBM SPSS Statistics 28 for descriptive analysis and group comparison. The objective of the qualitative in-depth interviews is to obtain a comprehensive picture of the personal experiences, motivations and support needs of members of the non-kin caregivers cohort, who are as heterogeneous as possible in terms of gender, socio-economic status, and facility with the German language. The qualitative data from the interviews will be examined using MAXQDA software, adopting a grounded theory approach for analysis.

**Discussion:**

This research will develop a comprehensive framework that captures the nuanced experiences of non-kin caregivers at the end of life. The framework will identify areas where support for non-kin caregivers is lacking and where further research is needed.

**Trial registration:**

The study was prospectively registered in the German Clinical Trials Register (Deutsches Register Klinischer Studien) (Registration N° DRKS00033889; date of registration: 05 April 2024). The study is searchable under the International Clinical Trials Registry Platform Search Portal of the World Health Organization, under the German Clinical Trials Register number.

## Introduction

### Problem area: Demographic developments and non-kin caregivers

The number of community-based initiatives on caring and compassionate communities is increasing in several countries across the globe [1,2]. Such initiatives take a public health approach to the provision of palliative care, aimed at strengthening family and non-kin caregiver networks [3]. Recent research on psychosocial cancer care suggests a shift in attention from the common model of a primary caregiving family member towards a broader, system-based concept of caregiving families and social networks [4–6]. Several rationales might be offered to explain this trend: First, there is greater awareness of the importance of dealing with illness, care needs, death, and grief, socially. Second, the number of single-person households is rising steadily (1996: 12.7 million; 2011: 15.4 million), with the 2021 German microcensus placing more than 16.6 million in this category. Of these, 35.6% are aged 65 years and older [7,8]. Demographers expect the number of elderly people with assistance and support needs to grow rapidly; at the same time, researchers predict that family members will become less capable of providing support [9]. These phenomena coincide with the general wish of people to live and die in a familiar environment [10]. Furthermore, for economic reasons, the German health care system prioritises outpatient care over inpatient treatment. Informal care is central in end-of-life situations; thus, private support networks may enable patients to remain in their own home environment at the end of a terminal illness situation, until death [11–13].

Individuals who live alone and lack relatives to take on caring and/or nursing tasks often depend on support and companionship from non-kin (e.g. friends, acquaintances, neighbours). Even individuals who live with a partner or spouse may depend on an extended informal network of caring friends and others [6]. Little is known about non-kin caregivers. Nonetheless, in Germany, 1 out of 10 patients receives support from only friends, acquaintances, and/or neighbours [14]. Moreover, an Austrian study indicated that non-kin often play a central role in private support networks [15].

In German society, non-kin caregivers are invisible [15]. They lack statistical presence, and they are not significantly addressed within political discourse or informational events. This is particularly surprising, giving that the priority of outpatient over inpatient care, as mandated in the German Social Welfare Code V (Sozialgesetzbuch [SGB] V), will presumably become more important over time. Care support and counselling centres for family caregivers (e.g. “Fachstellen für pflegende Angehörige” and “Pflegestützpunkte” in Germany) [16–18] do not yet offer services specifically for non-kin caregivers.

The proposed project aims at addressing the largely unknown contributions, experiences, and support needs of non-kin caregivers.

### Conceptual definition: Non-kin caregivers

Different conceptualisations of informal caregiving and informal caregivers exist. Unfortunately, there is no consensus over these definitions in the scientific community and amongst key stakeholders in the long-term care sector, including the World Health Organization, the United Nations, the Organisation for Economic Co-operation and Development, the International Alliance of Carer Organizations, and the European Association Working for Carers. This poses a challenge for the comparability of research results, also across countries [19,20]. Tur-Sinai [20] derived the following essential characteristics from the diverging definitions of informal caregivers in the literature: “someone who provides care (1) at least weekly (2) to someone with a chronic illness, disability, or other long-lasting health, social or long-term care needs, (3) as part of an unpaid non-contractual voluntary work outside a professional or formal framework.” This definition encompasses both next-of-kin and non-kin caregivers. The proposed project will focus on the sub-group of non-kin caregivers (e.g. neighbours, friends) who support individuals who are living alone at the end of life. This sub-group includes co- and extra-resident caregivers [21]. Non-kin caregivers may support and accompany patients in various ways, and to various extents. Small groups of caregivers may also form a caregiving network or a caring community in which they network with professional health care providers (e.g. palliative home care or meal service providers, family practitioners).

### Current state of research

The German Survey on Volunteering (Freiwilligensurvey) revealed that, in 2014, 3.4% of the German population aged ≥14 years voluntarily provided informal support to neighbours or friends with care needs [22]. Several systematic reviews of family caregivers’ unmet needs [23] and overviews of assessment tools for the needs of family caregivers [24] in palliative and advanced cancer care have revealed some knowledge of the family caregiver situation. Specifically, in Germany, family caregivers shoulder a heavy share of caregiving tasks [25]; and across Europe and worldwide, they provide a significant and often invisible amount of care [9,20,26], taking over such tasks as housekeeping, nursing, and organising care. The physical, financial, and mental burdens of this caregiving may lead to pathological conditions requiring treatment [27–30]. Commonly, family caregivers express the need for emotional support, disease-specific knowledge, role responsibilities, self-care, and general practical support [23]. Research has also revealed the benefits and positive aspects of informal caregiving, including a sense of meaning and feelings of accomplishment [31]. However, the literature on non-kin caregiver activities is scarce. Overall, such caregivers have received little attention in gerontological and palliative care research [15,32]. The few available studies indicate that friends are more willing than neighbours to take over physical caregiving tasks. Specifically, caring friends tend to assist with a greater number of tasks and invest more hours per week than neighbours; but they are also older, on average, than the latter [13,33].

### Research gaps

Given social trends, the topic of non-kin caregiving is likely to increase in relevance. Research is needed on the characteristics of non-kin caregivers, their care recipients, and their caregiving situations. Previous research has focused on the burdens and needs of family caregivers, as well as the benefits of caregiving. However, little is known about the specific burdens, needs, and benefits of non-kin caregivers, the extent and type of care they provide, and the manner in which they network. Furthermore, research is needed to determine which support offers they may require or desire. For instance, friends, neighbours, and other non-kin caregivers might welcome opportunities for exchange with others in similar situations and, at times, access to professional or formal support.

## Materials and methods

### Trial registration

The study was prospectively registered in the German Clinical Trials Register (Deutsches Register Klinischer Studien) (Registration N° DRKS00033889; date of registration: 05 April 2024). The study is searchable under the International Clinical Trials Registry Platform Search Portal of the World Health Organization, under the German Clinical Trials Register number.

### Study aim

The proposed project aims at gaining knowledge on: (1) non-kin extra-resident caregivers for individuals at the end of life, (2) non-kin caregivers’ contributions to end-of-life care, and (3) non-kin caregivers’ experiences and support needs.

### Study design

**Fig 1:**
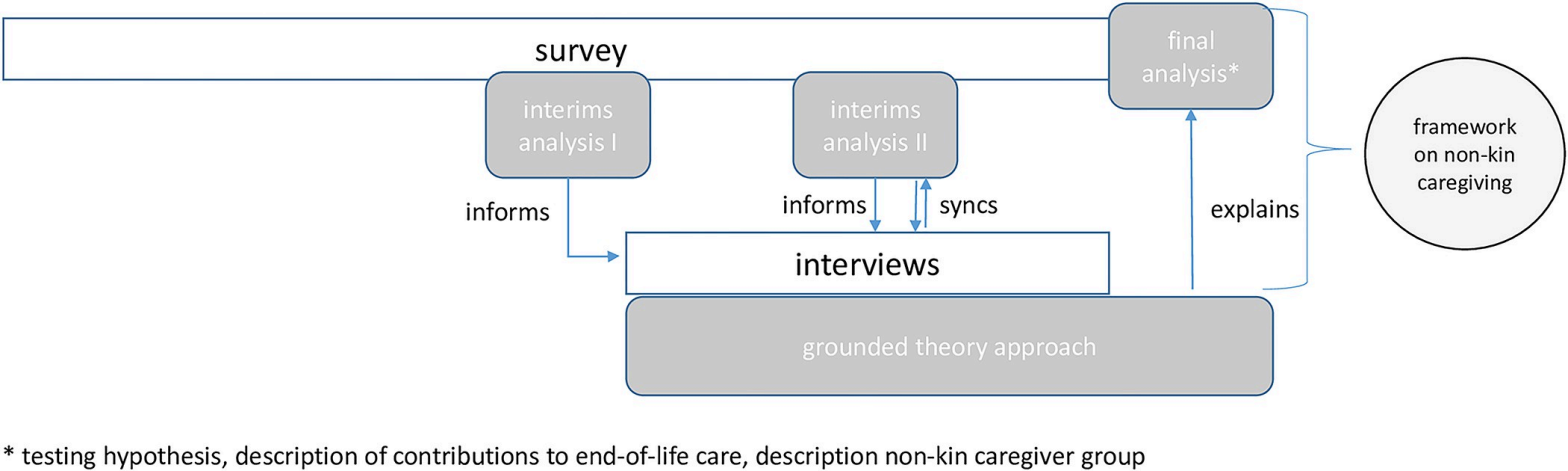
Research procedure: Explanatory sequential design.

The research will apply an explanatory, sequential, mixed-methods design [34]. Quantitative socio-demographic, caregiving, and survey data will be supplemented by qualitative interviews with non-kin caregivers, following an interim analysis (see Fig 1). The ability to draw from both qualitative data (applying a pragmatic, bottom-up, grounded theory approach [35,36]) and quantitative data will enable the construction of a broad conceptual framework of non-kin caregiving. The embedding of the quantitative and qualitative data in the interim analysis II will allow for further refinement of the recruitment strategy and interview guide, thereby enhancing the insights generated.

The core research questions will be: (a) Population: What are the characteristics of non-kin caregivers, their care recipients, and their caregiving situations? (b) Contributions to end-of-life care: To what extent (in terms of tasks and time) do non-kin caregivers contribute to care? How do non-kin caregivers differ from family caregivers, with respect to relationship quality and experienced closeness with the care recipient? How do non-kin caregivers ally with informal community-based support/caregiving networks and interact with formal caregiving structures? (c) Experiences and needs: What burdens and benefits arise from non-kin caregiving? Are there differences in the perceived burdens and benefits of caregiving within the diverse population of non-kin caregivers? What obstacles and conducive factors related to particular caregiving situations do non-kin caregivers experience (e.g. admission/discharge from hospital, initiation of formal care structures, death of the care recipient)?

### Study population, data collection and analyses

#### Quantitative survey

The survey will determine the characteristics of non-kin caregivers and their caregiving tasks, and measure the burdens and benefits of caregiving. Non-kin caregivers will be asked to complete an online survey consisting of four quantitative, validated surveys and one scale:

1. The 10-item Burden Scale for Family Caregivers, short version (BSFC-s) [37,38] will be used to assess subjective burden among non-kin caregivers. This measure requires respondents to rate their degree of subjective burden on a 4-point Likert-type scale ranging from 0 (strongly disagree) to 3 (strongly agree) (approx. duration: 3–5 min.).
2. The 14-item Benefits of Being a Caregiver Scale (BBCS) [39] will be used to assess participants’ experienced benefits of being a caregiver. This measure requires respondents to rate their subjective experiences on a 5-point Likert-type scale ranging from 0 (strongly disagree) to 4 (strongly agree) (approx. duration: 5 min.).
3. The 20-item Family Inventory of Needs (FIN) [40,41] will be used to measure the importance of the care needs of caregivers of advanced cancer patients and the extent to which these caregivers perceive that their care needs are being met. This measure requires respondents to rate the importance and fulfilment of each of 10 needs on a 10-point Likert-type scale ranging from 0 (not important) to 3 (very important) (approx. duration: 10 min.).
4. The 9-item Positive Mental Health Scale (PMH-scale) [42] will be used to assess participants’ positive mental health, combining emotional, psychological, and social aspects of well-being. This measure requires respondents to rate their degree of well-being on a 4-point Likert-type scale ranging from 0 (do not agree) to 3 (agree) (approx. duration: 5 min.).
5. The 2-item Graphic Closeness Scale (GCS) [43] will be used to assess participants’ relational intimacy in their current situation and prior to the care recipient’s illness on a one-dimensional line, with the study participant as the anchor point. The measure uses a response scale ranging from 1 (very close) to 0 (not close/very distant) (approx. duration: 2–3 min.).

The BSFC-s and BBCS were specifically developed for next-of-kin caregivers. However, individual survey items are formulated in such a way that they can be transferred to non-kin caregivers. In the proposed study, the quantitative analysis will provide an initial indication of the usability of these instruments for non-kin caregivers. A comparison will be drawn between the results for non-kin caregivers and those of previous studies on next-of-kin caregivers. For example, related to the GCS, Neyer et al. [43] hypothesised that relationships with non-kin caregivers would show lower levels of emotional closeness than next-of-kin relationships, as non-kin relationships might be primarily perceived in terms of reciprocity.

Socio-demographic and caregiving-related data will provide further background information on non-kin caregiver characteristics. The following socio-demographic and caregiving-related aspects are commonly reported in the literature and will be collected in our survey to enable comparison: age, gender, family/relationship status, ethnicity, religious preference, employment status, educational attainment, caregiver economic status, dwelling/household status, relationship to the care recipient, care recipient dwelling status, number of days/months/years of caregiving, and number of hours of care provided to the care recipient per week. Furthermore, the number of potential further non-kin caregivers and possible coordination between them, as well as possible coordination with professional services (e.g. nursing services) will be recorded. Also, the socio-demographic and caregiving-related questionnaire will be used to obtain data on written caregiving agreements and the receipt of remuneration. The collected socio-demographic and caregiving-related data will enable an (inter)national comparison to be drawn with other informal caregiver groups, based on findings reported in the literature.

Using items 27–34 of the Eurofamcare Common Assessment Tool [44], the following support dimensions will be recorded: (1) shopping, collecting prescriptions, and running other errands; (2) transporting or accompanying the patient to appointments; (3) cooking, cleaning, doing laundry, gardening, and performing other household chores; (4) maintaining and repairing the patient’s home; (5) washing, dressing, feeding, and assisting the patient in going to the toilet; (6) assisting with medication, medical treatment, and therapy; (7) making telephone calls, writing letters and/or e-mails, filling in forms; (8) managing finances; (9) providing financial support, accessing counselling and information about the patient’s illness and/or support services, and organising and managing care and support; and (10) providing emotional support, companionship, and reassurance. These aspects will be further investigated by questions relevant to the specific study population, investigating (for example) knowledge about patients’ end-of-life care preferences, existing proxies, and preferences and preparations concerning the time after death (e.g. burial or inheritance).

Inclusion criteria for the survey will be: Non-kin caregivers must be ≥18 years old and providing care at least weekly for at least 4 hours per week, as unpaid, non-contractual, voluntary work outside of a professional or formal framework [20]. Care-recipients must be ≥ 18 years old and suffering from a malignant or non-malignant life-limiting chronic disease.

According to the German Survey on Volunteering, 3.4% (approximately 2,435,400 persons) of the population aged ≥ 14 years provide informal care to neighbours or friends in Germany [22]. At the same time, the limited descriptive data on informal caregivers show that nearly 1 out of 10 caregivers provides extra-household non-kin care [25]. Considering the lead researchers’ previous recruitment experiences [45,46] and the planned recruitment strategy (see above), a convenience sample size of approximately 100–150 participants is expected, providing a comprehensive representation of the population of non-kin caregivers.

A quantitative socio-demographic assessment instrument to accompany the four validated quantitative surveys and one scale will be developed. A pretest will be performed with two to three non-kin caregivers to ensure the comprehensibility and appropriateness of the questions and the appropriateness of the length. The results of the pretest will be reviewed by the research team, the research advisory board and the project’s patient and public involvement (PPI) group in digital advisory board meeting. At the same meeting, the specifics of the recruitment strategy will be reviewed.

The recruitment period will commence on 1 December 2024 and conclude on 31 January 2026. The survey will be sent out by the project team to potential study participants named by recruitment partners, distributed via recruitment partners’ online distribution lists, and placed online by the project team. To mitigate the risk of a low response rate, the recruitment strategy will integrate national and regional public volunteering platforms, print media, social media, and caregiving and neighbourhood forums, and will involve committed participants from a previous study by A. Pendergrass [47]. A short recruitment video will be developed, to encourage participation and introduce the project to potential caregivers. A public website will also be built, containing information on: (1) the project in different languages (i.e. written and multilingual videos), (2) possibilities for participation (e.g. online survey link), and (3) contact persons. Study participants will be asked if they are willing to participate in the qualitative in-depth interviews. Participants’ contact data and information regarding possible interview participation will be stored separately from the questionnaires.

For the analysis, quantitative data will be entered into IBM SPSS Statistics 28. Quantitative data will be analysed according to existing manuals, and in comparison with normative data from standard German population samples. Prior to the main analyses, data cleaning and interim analyses will be conducted. Accurate data entry will be ensured through a series of steps, including item range evaluation and outlier checking. Response data will be examined for plausibility and missing data. Data accuracy will be double-checked. Descriptive statistics will be calculated to test normality assumptions in the relevant outcome variables. Frequencies and group comparisons according to the hypotheses will be performed.

During participant recruitment, especially for the interviews, participants will be asked for their motivation to participate in the project and potential participants who decline participation will be asked for their reasons. Data on the participation rate and reasons for (non-)participation will be recorded and entered into IBM SPSS Statistics 28. Recruitment data will be used to enhance the recruitment strategy, if necessary.

### Qualitative interviews

Qualitative in-depth interviews will be carried out with non-kin caregivers, with the aim of generating a rich picture of the complex phenomenon of non-kin caregiving with in-depth descriptions of caregivers’ experiences, contributions to end-of-life care, and support needs.

The results of a scoping review, currently conducted at the Institute for General Practice and Palliative Care, Hannover Medical School, the research questions, additional questions contextualising the hypothesis, and the project PPI group and advisory board will inform the development of the interview guide. Questions will fall into the domains of non-kin caregivers’ contributions to end-of-life care, experiences, and needs. Qualitative interviews will include in-depth information on the impact of the kind of caregiving relationship (e.g. friend vs. neighbour, primary vs. secondary caregiver, part of a community/neighbourhood network vs. not in such a network) and the care recipient’s situation (e.g. living alone vs. with family, cared for by a friend vs. a neighbour, having one vs. several informal caregivers) on caregiving tasks, benefits, and burdens. The interview guide will be pretested with two PPI group members for appropriateness, comprehensibility, and feasibility, and adapted accordingly.

Individual interviews with non-kin caregivers will be conducted. The recruitment period will commence on 1 December 2024 and conclude on 31 January 2026. According to research methodology guidelines, at least 12 interviews are typically required to achieve relative qualitative data saturation [48]. However, in this study, a larger number of interviews may be needed, as non-kin caregivers present heterogeneous characteristics in terms of the type of relationship with the care recipient, gender, age, employment situation, socio-economic status, and urban/rural residence. With recourse to “information power” [49], considering the study objective, sample specificity, theoretical background, quality of interview dialogue, and analysis strategy, the sample size will be extended to 20–25 non-kin caregivers, if needed. The inclusion criteria of the survey will be applied to the study participants, and participants in the quantitative survey will be asked if they consent to follow-up contact by the project team with regard to a qualitative, in-depth interview. To achieve maximal diversity among the interview participants, the research team will purposefully select individuals willing to participate according to the abovementioned diversity criteria. Socio-demographic data from the survey will provide background information on the diversity of non-kin caregivers, and efforts will be made to include caregivers from diverse communities (e.g. non-native German speakers). All participating non-kin caregivers whose mother tongue is not German will be offered an interpreter. In the event of a lack of eligible individuals or a lack of diversity of individuals consenting to participation, the research team will apply purposive sampling and actively recruit individuals who fulfil the diversity criteria. If necessary, further strategies will be considered, such as advertisements in newspapers and social media posts.

All qualitative interviews will be voice-recorded and transcribed verbatim by the research assistant, using the transcription software f4 (dr. dresing & pehl GmbH, Marburg, Germany). The collected data will be continuously analysed. Interview transcripts will be analysed based on a pragmatic, bottom-up, grounded theory approach [35,36], aimed at developing an initial framework of non-kin caregivers’ contributions to end-of-life care, experiences, and needs, using the qualitative data analysis software MAXQDA (VERBI GmbH, Berlin, Germany). Two researchers of the research team will independently code the first three interview transcripts; ongoing coding will be done by one researcher. After an assumed 50% of the interviews (approx. 12) are coded, a consistency check of the delimitation of categories and coherence of internal category codings will be performed; this will be repeated when the coding of all interviews is complete. In the first step, meaningful units of text will be openly coded through an inductive process that develops and assigns descriptive codes. In the second step, codes with similar content will be grouped under concepts representing core topics. The project team will discuss the declaration of meaningful units and the definition of concepts. Similar concepts will be merged into broader categories using an axial coding strategy. Throughout the coding process, theoretical memos will be written to define codes, concepts, and categories and to trace the development of ideas. This preliminary data analysis, running parallel to the data collection, will be used to determine when relative data saturation and information power are reached; at this point, the data collection will be terminated [49].

### Quantitative and qualitative data embedding

In the sequential, exploratory design, the quantitative and qualitative data will be integrated through the sampling procedure and by building the qualitative data collection approach on the interim analysis of the quantitative data. The data analysis will merge all quantitative and qualitative data sets, resulting in a conceptual framework of non-kin caregiving [50].

Online team meetings will be held regularly to merge the qualitative and quantitative data analysis, developing a meaning-centred approach, aside from the variable-centred analysis of socio-economic data and support dimensions. Quantitative data will be discussed according to contextual aspects (interim analysis II). Emerging questions will be transferred to the analysis of the qualitative material and will inform an adaption of the interview guide within a joint PPI group and advisory board meeting. The qualitative data will be analysed for explanations and in-depth insights (interim analysis II), with reference to the study hypotheses. The heterogeneous sample will provide a range of perspectives, and data evaluation will consider participants’ origins, values, norms, interests, and motivations, to capture subjective perspectives on non-kin caregiving and integrate these into a holistic picture.

### Schedule/Course of study per participant

The total duration of the study will be 24 months. The duration of particiation on the survey study will be approximatly 25–28 minutes. All individuals who participate in the survey will be invited to participate in an interview, which will take between 45 and 90 minutes.

### Patient and public involvement

The proposed study design was developed on the basis of feedback from a non-kin caregiver of a frail neighbour for several years, until the neighbour’s death. The caregiver reported that she would like research to address the motivations for non-kin caregiving and the patient’s family situation. Over the course of the study, patients and public representatives will be invited to voluntarily serve as consultants. The PPI group will be as diverse as possible, including members that are heterogeneous with regard to gender, socio-economic status, and facility with the German language. Depending on an agreement on joint values at the beginning of the project (e.g. using a partnership canvas), the PPI group could be tasked with: (1) consulting and providing feedback on the development of the interview guide, (2) pretesting the survey and interview guide for appropriateness and feasibility, in order to improve comprehensiveness and mitigate the risk of burden, (3) providing feedback on the comprehensibility of the study information and consent form, (4) supporting access to their community, (5) reflecting on the study results and the developed framework on non-kin caregiving, and (6) disseminating the project findings to lay readers.

### Quality assurance

Hudson et al.’s [51] self-assessment instrument for designing and conducting palliative care research with family carers will be used to enhance methodological rigour. Reporting guidelines for participation (GRIPP II [52]), qualitative research (COREQ [53]), and surveys (CHERRIES [54]) will be implemented.

Torensma et al.’s [55] self-assessment instrument, “Diversity Responsiveness in Palliative Care Projects”, will be administered throughout the project to ensure responsiveness to diversity issues. Throughout the data analysis, the material will be checked for diversity aspects regarding non-kin caregiver characteristics and the caregiver–patient relationship. The project will use interpreters to facilitate the inclusion of neglected groups and vulnerable populations (e.g. non-kin caregivers with an immigrant background). The project team aims at establishing a diverse research team and PPI group, with respect to age, gender, and socio-cultural background.

A multifaceted advisory board will support the research and project team throughout the project duration, providing valuable input to differentiate the findings from those of previous research on other groups and contexts (e.g. family caregivers of end-of-life patients), thereby increasing the relevance of the research for clinical practice and facilitating greater outreach. Specifically, the advisory board will support the project team in: (1) reviewing the project design and methodology, (2) discussing project progress, (3) refining the recruitment strategy, (4) reflecting on the project results and the developed model of non-kin informal caregiving, and (5) supporting the dissemination of the project results. Furthermore, the advisory board will provide constructive advice on the recruitment of underrepresented groups. The board will enhance the relevance, quality, and acceptance of the research project by co-shaping the project and advising on the prioritisation of the results. The board will include national and international researchers on end-of-life care with expertise in (non-kin) caregiver research; (inter)national researchers with expertise in mixed methods; and hospice and palliative care practitioners (i.e. physicians, nurses, case managers, counsellors, social workers, psychologists, psycho-oncologists).

The study has been approved by the Ethics Committee of the Hannover Medical School (13.04.23) No. 10857_BO_K_2023. The Ethics Committee of the Friedrich-Alexander-Universität Erlangen-Nürnberg has stated that there is no need for further consultation, as the research project has already been reviewed by an Ethics Committee established under state law (19.04.2002) No. 23-105-Bn. Participants in the study will give written informed consent to participate in the study.

## Discussion

### Expected results

The findings of the embedded quantitative and qualitative data and the preliminary literature review will inform the creation of a conceptual framework. This framework will show the prerequisites and circumstances of non-kin caregiving, as well as non-kin caregivers’ contributions to end-of-life care, experiences and dependencies within the caregiving situation, relationships with patients, mental well-being and caregiving tasks, and support needs. The framework will include conceptualisations and associations, and will highlight obstacles and conducive factors for non-kin caregiving for patients at the end-of-life. Thus, the framework will outline the phenomenon of non-kin caregiving and guide the development of support measures for non-kin caregivers and new research questions.

### Dissemination of the research results

Once the data analysis is complete and written up, manuscripts will be published in peer-reviewed open-access journals. Furthermore, the results will be presented at (inter)national conferences in the fields of end-of-life care, health services research, and general practice. Data files with NO personally identifiable information will be maintained after the study conclusion. In accordance with the DFG code of conduct, “Guidelines for Safeguarding Good Research Practice” [56], the project team will disseminate the research results in a timely manner and will not withhold unidentifiable data from other professionals seeking to verify the conclusions made. Educational workshops for inpatient and community palliative care services will be administered to communicate the main project results.

## Supporting information

S1 Checklist STROBE statement—Checklist of items that should be included in reports of observational studies. (DOCX)
