# Supplementary material for: Non-kin caregivers of terminally ill people: Contributions, experiences, and needs: A protocol for a mixed-methods study
Source: PLoS One. 2024 Jun 27;19(6):e0306282. doi: 10.1371/journal.pone.0306282 (PMC11210750; doi:10.1371/journal.pone.0306282)
Supplement: S1 Checklist — (DOCX) [file pone.0306282.s001.docx]

STROBE Statement—checklist of items that should be included in reports of observational studies

|  | Item No. | Recommendation | Page  No. | Relevant text from manuscript |
| --- | --- | --- | --- | --- |
| **Title and abstract** | 1 | (*a*) Indicate the study’s design with a commonly used term in the title or the abstract | 1 | Non-kin caregivers of terminally ill people: Contributions, experiences, and needs: a protocol for a mixed-methods study |
|  |  | (*b*) Provide in the abstract an informative and balanced summary of what was done and what was found | 2-3 | Abstract |
| Introduction | | | |  |
| Background/rationale | 2 | Explain the scientific background and rationale for the investigation being reported | 4-7 | Introduction |
| Objectives | 3 | State specific objectives, including any prespecified hypotheses | 7 | Study aim |
| Methods | | | |  |
| Study design | 4 | Present key elements of study design early in the paper | 7-8 | Design |
| Setting | 5 | Describe the setting, locations, and relevant dates, including periods of recruitment, exposure, follow-up, and data collection | 8-16 | Study population, data collection and analyses |
| Participants | 6 | *Cross-sectional study*—Give the eligibility criteria, and the sources and methods of selection of participants | 10+12 | Study population and data collection, Inclusion and exclusion criteria |
| Variables | 7 | Clearly define all outcomes, exposures, predictors, potential confounders, and effect modifiers. Give diagnostic criteria, if applicable |  | n/a |
| Data sources/ measurement | 8* | For each variable of interest, give sources of data and details of methods of assessment (measurement). Describe comparability of assessment methods if there is more than one group | 8-14 | Study population and data collection (survey and interviews) |
| Bias | 9 | Describe any efforts to address potential sources of bias | 15 | Torensma et al.’s self-assessment instrument, “Diversity Responsiveness in Palliative Care Projects”, will be administered throughout the project to ensure responsiveness to diversity issues. Throughout the data analysis, the material will be checked for diversity aspects regarding non-kin caregiver characteristics and the caregiver–patient relationship. The project will use interpreters to facilitate the inclusion of neglected groups and vulnerable populations (e.g. non-kin caregivers with an immigrant background). The project team aims at establishing a diverse research team and PPI group, with respect to age, gender, and socio-cultural background. |
| Study size | 10 | Explain how the study size was arrived at | 10 + 12 | Survey: The limited descriptive data on informal caregivers show that nearly 1 out of 10 caregivers provides extra-household non-kin care. Considering the lead researchers’ previous recruitment experiences and the planned recruitment strategy, a convenience sample size of approximately 100–150 participants is expected, providing a comprehensive representation of the population of non-kin caregivers.  Interviews: With recourse to “information power”, considering the study objective, sample specificity, theoretical background, quality of interview dialogue, and analysis strategy, the sample size will be extended to 20–25 non-kin caregivers, if needed. The inclusion criteria of the survey will be applied to the study participants, and participants in the quantitative survey will be asked if they consent to follow-up contact by the project team with regard to a qualitative, in-depth interview. |

Continued on next page

| Quantitative variables | 11 | Explain how quantitative variables were handled in the analyses. If applicable, describe which groupings were chosen and why | 11 | Data analysis (survey) |
| --- | --- | --- | --- | --- |
| Statistical methods | 12 | (*a*) Describe all statistical methods, including those used to control for confounding | 11 | Data analysis (survey) |
|  |  | (*b*) Describe any methods used to examine subgroups and interactions | 11 | Data analysis (survey) |
|  |  | (*c*) Explain how missing data were addressed | 11 | Data analysis (survey) |
|  |  | *Cross-sectional study*—If applicable, describe analytical methods taking account of sampling strategy | 11 | Data analysis (survey) |
|  |  | (*e*) Describe any sensitivity analyses |  | n/a |
| Results | | | | |
| Participants | 13* | (a) Report numbers of individuals at each stage of study—eg numbers potentially eligible, examined for eligibility, confirmed eligible, included in the study, completing follow-up, and analysed |  | n/a (study protocol) |
|  |  | (b) Give reasons for non-participation at each stage |  | n/a (study protocol) |
|  |  | (c) Consider use of a flow diagram |  | n/a (study protocol) |
| Descriptive data | 14* | (a) Give characteristics of study participants (eg demographic, clinical, social) and information on exposures and potential confounders | 10-11 | Study population and data collection, Socio-demographic and caregiving-related data |
|  |  | (b) Indicate number of participants with missing data for each variable of interest |  | n/a (study protocol) |
| Outcome data | 15* | *Cross-sectional study—*Report numbers of outcome events or summary measures |  | n/a |
| Main results | 16 | (*a*) Give unadjusted estimates and, if applicable, confounder-adjusted estimates and their precision (eg, 95% confidence interval). Make clear which confounders were adjusted for and why they were included |  | n/a (study protocol) |
|  |  | (*b*) Report category boundaries when continuous variables were categorized |  | n/a (study protocol) |
|  |  | (*c*) If relevant, consider translating estimates of relative risk into absolute risk for a meaningful time period |  | n/a (study protocol) |

Continued on next page

| Other analyses | 17 | Report other analyses done—eg analyses of subgroups and interactions, and sensitivity analyses | 11 | Data analysis |
| --- | --- | --- | --- | --- |
| Discussion | | | | |
| Key results | 18 | Summarise key results with reference to study objectives | 16 | Expected results (study protocol) |
| Limitations | 19 | Discuss limitations of the study, taking into account sources of potential bias or imprecision. Discuss both direction and magnitude of any potential bias |  | n/a |
| Interpretation | 20 | Give a cautious overall interpretation of results considering objectives, limitations, multiplicity of analyses, results from similar studies, and other relevant evidence |  | n/a (study protocol) |
| Generalisability | 21 | Discuss the generalisability (external validity) of the study results |  | n/a (study protocol) |
| Other information | |  | | |
| Funding | 22 | Give the source of funding and the role of the funders for the present study and, if applicable, for the original study on which the present article is based |  | Funding (submission portal as required from PLOS ONE) |

*Give information separately for cases and controls in case-control studies and, if applicable, for exposed and unexposed groups in cohort and cross-sectional studies.

**Note:** An Explanation and Elaboration article discusses each checklist item and gives methodological background and published examples of transparent reporting. The STROBE checklist is best used in conjunction with this article (freely available on the Web sites of PLoS Medicine at http://www.plosmedicine.org/, Annals of Internal Medicine at http://www.annals.org/, and Epidemiology at http://www.epidem.com/). Information on the STROBE Initiative is available at www.strobe-statement.org.
